# Persimmon Leaves (*Diospyros kaki*) Extract Protects Optic Nerve Crush-Induced Retinal Degeneration

**DOI:** 10.1038/srep46449

**Published:** 2017-04-20

**Authors:** Hong Ryul Ahn, Kyung-A Kim, Suk Woo Kang, Joo Young Lee, Tae-Jin Kim, Sang Hoon Jung

**Affiliations:** 1 Natural Products Research Center, Korea Institute of Science and Technology (KIST), Gangneung 25451, Republic of Korea; 2 Department of Biological Chemistry, Korea University of Science and Technology (UST), Daejeon 34113, Republic of Korea; 3 Department of Biological Sciences, Pusan National University, Busan 46241, Republic of Korea

## Abstract

Retinal ganglion cell (RGC) death is part of many retinal diseases. Here, we report that the ethanol extract of *Diospyros kaki* (EEDK) exhibits protective properties against retinal degeneration, both *in vitro* and *in vivo*. Upon exposure to cytotoxic compounds, RGC-5 cells showed approximately 40% cell viability versus the control, while pre-treatment with EEDK markedly increased cell viability in a concentration-dependent manner. Further studies revealed that cell survival induced by EEDK was associated with decreased levels of apoptotic proteins, such as poly (ADP-ribose) polymerase, p53, and cleaved caspase-3. In addition to apoptotic pathways, we demonstrated that expression levels of antioxidant-associated proteins, such as superoxide dismutase-1, glutathione S-transferase, and glutathione peroxidase-1, were positively modulated by EEDK. In a partial optic nerve crush mouse model, EEDK had similar ameliorating effects on retinal degeneration resulting from mechanical damages. Therefore, our results suggest that EEDK may have therapeutic potential against retinal degenerative disorders, such as glaucoma.

As a light-sensitive tissue responsible for vision, the retina converts visual information into electrical signals that travel to the brain through the optic nerve^1^. Although the retina occupies only a small portion of the central nervous system, it is among the body’s highest oxygen-consuming tissues and comprises specialized neurons, including photoreceptors and retinal ganglion cells (RGCs)^2^.

Increased oxygen consumption can therefore give rise to oxidative stress-induced damage to the retina, which in turn could be a potential risk factor for several retinal disorders^3^,^4^,^5^,^6^. Glaucoma is one of the major sight-threatening diseases and is known to damage the optic nerve, a bundle of RGC axons of the retina. Glaucoma is closely associated with increased intraocular pressure (IOP)^7^,^8^. Currently, therapies for glaucoma are designed to reduce the IOP to delay the progression of visual loss^9^. However, glaucoma patients who have been treated with IOP-lowering medications can sometimes still progress, and patients with normal IOPs can occasionally develop glaucomatous optic neuropathy, varying based on age and ethnicity^10^. Further, progressive axonal deterioration in RGCs caused by a variety of factors, such as abnormal IOP, high production of free radicals, and failure of antioxidant defence, may be the multifactorial cause of RGC death that subsequently results in visual field defects^11^,^12^,^13^. Accordingly, protecting RGCs is one of the central therapeutic challenges in preventing the pathogenesis of glaucoma, and considerable efforts in basic and clinical research have been made to mitigate RGC degeneration to date^14^,^15^,^16^,^17^.

The leaves of persimmon, *Diospyros kaki* Thunberg (Ebenaceae) are most widely cultivated in countries in eastern Asia, including China, Korea, and Japan. Previous data have shown that *D. kaki* contains abundant bioactive chemicals, such as flavonoids, polyphenols, organic acids, and vitamins, which could contribute to their pharmacological characteristics, such as their potent radical-scavenging and antioxidant properties^18^,^19^. Not surprisingly, some evidence suggests that the leaves of *D. kaki* show medicinal effects against haemostasis, diuresis, constipation, and hypertension^20^,^21^. In addition, two reports have shown that components of *D. kaki* have beneficial effects on eye diseases in humans^22^,^23^. Nevertheless, it remains questionable whether *D. kaki* leaves are useful in preventing and treating retinal disorders.

The aim of this study was therefore to determine whether the leaves of *D. kaki* have protective effects on retinal degeneration induced by oxidative stress and optic nerve damage.

## Methods

### Chemicals

High-grade solvents were utilized for extraction. Hoechst 33342 and propidium iodide (PI) were obtained from Molecular Probes (Eugene, OR, USA). Antibodies against poly (ADP-ribose) polymerase (PARP), cleaved caspase-3, p53, apoptosis-inducing factor (AIF), and glutathione S-transferase (GST) were purchased from Cell Signaling Technology (Beverly, MA, USA). Superoxide dismutase-1 (SOD-1), glutathione peroxidase-1 (GPx-1), β-actin, and HRP-conjugated goat IgG antibodies were purchased from Abcam (Cambridge, UK). All other chemicals and reagents were purchased from Sigma-Aldrich (St Louis, MO, USA).

### Plant materials

*D. kaki* leaves were collected in Gangneung, Gangwon Province of Korea in August 2013. A voucher specimen (D-521) was deposited in the Korea Institute of Science and Technology (KIST) Gangneung Institute to ensure long-term care and maintenance of the sample. Dried leaves of *D. kaki* were extracted 3 times with ethanol at room temperature in an ultrasonic cleaning bath (model RK 158 s, Bandelin, Germany)^24^.

### Animals

All animal studies were conducted in a pathogen-free barrier zone at the KIST Gangneung Institute and were performed in accordance with the procedure outlined in the Association for Research in Vision and Ophthalmology Statement for the Use of Animals in Ophthalmic and Vision Research. Procedures used in this study were approved by the Animal Care and Use Committee of KIST (Approval No. 2014-011).

In the present study, male C57BL/6 J mice weighing between 20–25 g (6 weeks of age, Central Lab. Animal Inc., Seoul, Korea) were used to study the protective effects of EEDK on partial optic nerve crush (PONC)-induced retinal damage. The mice were acclimated for 1 week, caged in groups of 8 mice, and had access to animal chow and water *ad libitum.* The mice were housed at 23 ± 0.5 °C and 10% humidity, with a 12-h light-dark cycle.

### Cell culture and cell viability

The transformed retinal ganglion cell line (RGC-5) was obtained from Alcon Research, Ltd. RGC-5 cells were seeded at 5.0 × 10^3^ cells/well in 96-well plates and incubated for 24 h, after which they were exposed to Dulbecco’s Modified Eagle’s Medium containing 1% foetal bovine serum plus EEDK or the vehicle. After a 1 h pre-treatment with various concentrations of EEDK, glutamate (10 mM) plus 1-buthionine-(S,R)-sulfoximine (BSO) (0.5 mM) (glutamate/BSO) was added to the cultures. To test cell viability, MTT (3-(4,5-dimethylthiazol-2-yl)−2,5-diphenyltetrazolium bromide) solution was added to the cells in plates (final concentration of 0.5 mg/ml) for 1 h at 37 °C. The optical density of the solubilized formazan product was measured using a spectrophotometer (BioTek Instruments, VT, USA) with a 570-nm test wavelength and a 690-nm reference wavelength.

### Microscopic analysis with PI and Hoechst 33342 double staining

The cells were stained with Hoechst 33342 (8 μM) with or without PI (1.5 μM) for 30 min at 37 °C to measure apoptotic or necrotic cell death caused by glutamate plus BSO^25^,^26^. After being washed twice, the cells were imaged using a fluorescence microscope (Olympus, Tokyo, Japan). PI-positive cells were counted using a cell counter under a fluorescence microscope at 100× magnification, and 4 representative images were used to estimate the percent of PI-positive cells and the total cell numbers (a minimum of 200 cells/well was counted).

### Assessment of reactive oxygen species (ROS) production

Cells were assessed for their production of ROS using the dye dihydroethidium (DHE), as described previously^27^,^28^. The cells were stained with DHE (10 μg/ml) on coverslips during a 30-min incubation at 37 °C in culture medium in a humidified chamber, after which they were fixed with 4% paraformaldehyde for 20 min. After washing in phosphate-buffered saline (PBS) containing 8 g/l, 0.2 g/l KCl, 1.44 g/l Na_2_HPO_4_, and 0.24 g/l NaH_2_PO_4_ (pH 7.4), coverslips were mounted in mounting medium (Dako, CA, USA), and red fluorescence was detected using a laser-scanning confocal microscope (Leica TCS SP5; Leica, Germany). The excitation wavelength was set at 514 nm and a 590-nm emission filter was used for fluorescence measurements. Image quantification was performed using Leica Application Suite 2.02 software, with 4 representative fields from each of 4 wells being sampled per group (n = 16 in each group).

### Immunocytochemistry

RGC-5 cells were grown on coverslips and fixed with 4% paraformaldehyde and blocked with blocking solution (1% BSA, 22.52 mg/ml glycine in PBST) for 1 hour at room temperature. The blocking buffer was removed, and the coverslips were washed three times with 1X PBST, before the addition of the primary antibody (Thy1.1 monoclonal antibody; Cell Signaling, Brn3a monoclonal antibody; Abcam, 1/500 dilution) with 1% BSA in PBST. The incubation was performed overnight at 4 °C. Coverslips were washed three times, 1:500 of secondary antibody (Alexa Fluor 488; Invitrogen-Molecular Probes) was added, and the coverslips were incubated for 1 hour in the dark. After the incubation, the coverslips were washed again three times with 1X PBST. The coverslips were mounted on glass slides (Prolong Gold antifade reagent with DAPI; Invitrogen). The cells were viewed with a fluorescence microscope (TE2000-U; NIKON, JAPAN).

### Protein extraction from cultured cells

RGC-5 cells were scraped using a cell scraper and centrifuged at 14,000 × *g* for 10 min. The cell pellets were lysed in cell lysis buffer (1 M Tris pH 7.4, 2 M NaCl, 1 M EDTA, 10% NP40, 1× protease inhibitors, 1 mM phenylmethylsulfonyl fluoride [PMSF]) and then incubated on ice for 10 min. The cell lysate was sonicated and centrifuged at 14,000 × *g* for 30 min at 4 °C.

### Protein extraction from mouse retinas

Retinal tissues were surgically dissected from mice and washed in cold PBS. Tissues were homogenized in RIPA buffer (150 mM NaCl, 1.0% IGEPAL CA-630, 0.5% sodium deoxycholate, 0.1% sodium dodecyl sulphate [SDS], 50 mM Tris, pH 8.0, 1× protease inhibitors, and 1 mM PMSF) and centrifuged at 14,000 × *g* for 30 min at 4 °C.

### Western blot analysis

Total protein concentrations were determined using a Bio-Rad Protein Assay Kit (Bio-Rad Laboratories, Hercules, CA, USA). Proteins (10 μg/lane) were loaded on a 10% SDS-polyacrylamide gel electrophoresis gel and then transferred to a polyvinylidene difluoride membrane (Hybond-P; Amersham Biosciences, GE Healthcare, UK).

Membranes were incubated with the following primary antibodies (each diluted 1:1,000): anti-PARP, anti-cleaved caspase-3, anti-p53, anti-AIF, anti-GST, anti-SOD-1, anti-GPx-1, and anti-β-actin. The membranes were washed with PBST (8 g/l NaCl, 0.2 g/l KCl, 1.44 g/l, Na_2_HPO_4_, 0.24 g/l, NaH_2_HPO_4_, and 0.1%Tween 20) and incubated with appropriate secondary antibodies (diluted 1:3,000) at room temperature for 2 h. Immunoreactive bands were detected using the enhanced chemiluminescence reagents (Amersham Bioscience, GE Healthcare, UK) and measured densitometrically using an LAS-4000 image reader and Multi Gauge 3.1 software (Fuji Photo Film, Japan).

### Histological analysis

Enucleated eyes were fixed in 10% formalin for 24 h, embedded in paraffin, and sectioned through an equatorial plane at a 4-μm thickness using a HM340E microtome (Walldorf, Germany). Briefly, haematoxylin solution (0.1% haematoxylin plus 10% ammonium) was added to the retinal section for 8 min. The sections were then washed 3 times with distilled water. Bluing reagent (0.2% lithium carbonate solution) was added to the sections for 1 min. The sections were quickly rinsed in 95% alcohol, and 1% Eosin Y solution was added to the sections for 1 min. Eosin Y was washed away with 95% alcohol 3 times, and the sections were coverslipped with a mounting medium and observed under a light-microscope (Olympus, Tokyo, Japan).

### PONC experiments

Mice were anesthetized by intraperitoneal injection of a mixture of Zoletil (1.6 μg/g; Virbac Laboratories 06515, France) and Rompun (0.05 μL/g, Bayer), and retinal damage was induced by PONC, as described. The optic nerve of the left eye was exposed by opening the meninges of the optic nerve with the sharp tips of a forceps (Jeung-do Bio & Plant Co., Ltd., Seoul, Korea), followed by blunt dissection. PONC was performed 2 mm behind the globe for 7 s with cross-action calibrated forceps (B-1, 00462 V, S&T AG, Switzerland). Various concentrations of EEDK were orally administrated before PONC, and the animals were scarified on day 7 after the PONC. The eyeballs were enucleated immediately and were used for retrograde labelling of RGCs.

### RGC labelling and retinal flat-mount preparation

Mice were anesthetized as described above, and a 5% solution of the neurotracer dye Fluoro-Gold (Invitrogen, NY, USA) was applied to their superior colliculi using a piece of soaked Gelfoam. Skull openings were then sealed with a petrolatum-based antibiotic ointment. The overlying skin was sutured and antibiotic ointment was applied externally. Seven days after the application of Fluoro-Gold, the eyes were enucleated, and the retinas were detached at the ora serrata and cut with a trephine around the optic nerve head. Four radial relaxing incisions were made, and the retinas were prepared as flattened whole mounts on silane-coated microscope slides^29^. Fluoro-Gold-labelled RGCs in flat-mounted retinas were counted in 4-mm^2^ areas, for a total of 16 fields of cells per retina (each field, 500 × 500 μm^2^) without position-matching.

### Statistical analysis

The data are expressed as the mean percentage of the control value plus the standard error of the mean (SEM). Statistical comparisons were made using one-way analysis of variance, followed by Dunnett’s test. Statistical analyses were conducted using GraphPad Prism, version 6.0 (GraphPad, San Diego, CA, USA). Differences were considered statistically significant at P < 0.05.

## Results and Discussion

### Effect of EEDK on RGC-5 cell death caused by glutamate/BSO-induced toxicity

It is well known that glutamate is the major excitatory neurotransmitter present in mammalian retinas, and excessive stimulation of *N*-methyl-d-aspartate receptors by glutamate could be a primary cause of neuronal cell death due to calcium overloading^12^,^30^. In addition to glutamate excitotoxicity, increased oxidative stress can also promote retinal cell death by necrotic and/or apoptotic signalling pathways, all of which have been implicated in the development of retinal degeneration^5^,^6^.

In this study, we investigated whether and how EEDK could play a role in retinal protection in response to excessive oxidative stress and excitotoxicity. Due to the unavailability of immortalized retinal cells for screening purposes, we used RGC-5 cells to study the beneficial effects of EEDK in our *in vitro* assays. Data from recent studies revealed that RGC-5 cells originated from a mouse (rather than a rat) and are not transformed retinal ganglion cells, but are more likely to be neuronal precursor cells^31^,^32^,^33^,^34^. Previously, we also verified the identify of RGC-5 cells by performing mitochondrial DNA sequencing, which demonstrated the murine origin of this cell line, and by showing similar responses to glutamate treatment compared with an earlier passage of the RGC-5 cell line^13^. Although there is controversy with respect to the experimental use of RGC-5 cells and its identity - some believe these cells to be 661 W cells; while others do not^35^, it may still be a useful tool for initial *in vitro* screening because RGC-5 appears to express RGC-typical proteins such as THY1 and BRN3, as well as neuronal markers^36^. We also confirmed that RGC-5 cells used in this study clearly expressed THY1 and BRN3a proteins through immunocytochemistry (Supplementary Fig. 1).

**Figure 1:**
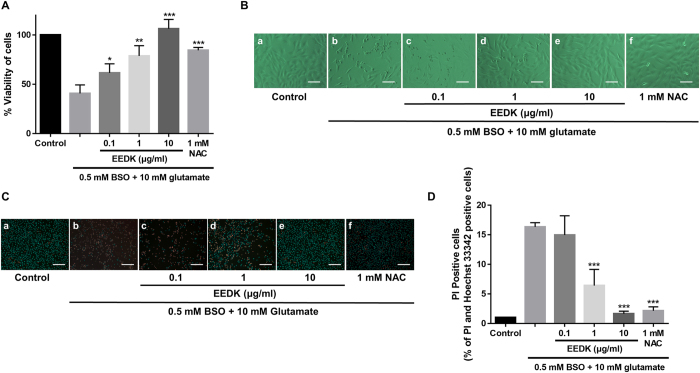
Effect of EEDK on RGC-5 cell death caused by glutamate/BSO-induced toxicity. (**A**) Effect of EEDK on the viability of RGC-5 cells exposed to 10 mM glutamate plus 0.5 mM BSO for 24 h, as measured in MTT assays. *N*-acetyl-l-cysteine (NAC; 1 mM) was used as a positive control. (**B**) Representative cell-morphology images obtained by light-contrast microscopy: (a) control cells (treated with 0.5% DMSO), (b) cells treated with 10 mM glutamate plus 0.5 mM BSO, (c–e) cells treated with EEDK (0.1 to 10 μg/ml), glutamate, and BSO, and (f) cells treated with 1 mM NAC with glutamate plus BSO. Scale bar = 50 μm. (**C**) Representative fluorescence images of PI (red) and Hoechst 33342 (blue) staining: (a) control cells (0.5% DMSO), (b) cells treated with 10 mM glutamate plus 0.5 mM BSO, (c–e) cells treated with EEDK (0.1 to 10 μg/ml), glutamate, and BSO, and (f) cells treated with 1 mM NAC with glutamate and BSO. Scale bar = 50 μm. Bar graphs represent the quantitative analysis of PI-positive cells. The results shown are the mean values with error bars indicating the S.E.M. (*p < 0.05, **p < 0.01, and ***p < 0.001). Experiments were repeated 3 times, independently.

As shown in Fig. 1, our results revealed that glutamate/BSO treatment significantly decreased cell viability, while the addition of EEDK under the same experimental conditions dramatically increased the survival rate of RGC-5 cells (Fig. 1A). The antioxidant *N*-acetyl-l-cysteine (NAC, 1 mM) was used as a positive control to evaluate the efficacy of EEDK. During cell death, bleb formation occurs at the plasma membrane, and subsequent rupturing can lead to an altered cell morphology that is easily detected^37^. Accordingly, cell-morphology changes were compared among the groups, and 10 μg/ml EEDK-treated cells showed inhibition of morphological deterioration resulting from glutamate/BSO-induced cellular damage (Fig. 1B). Further observations were made following double staining with Hoechst 33342 and PI, which are frequently used for simultaneous fluorescence-imaging analysis. Hoechst 33342 is a well-characterized blue fluorescent dye that can enter live cells and bind the minor groove of A/T-rich double-stranded DNA sequences^38^. In contrast, PI is a red membrane-impermeable dye that is commonly used to identify dead cells^39^. We observed that pre-treatment with EEDK clearly attenuated the detection of red-stained nuclei in a concentration-dependent manner (Fig. 1C,D), suggesting that EEDK can play a protective role against RGC-5 cell death caused by excitotoxicity and oxidative stress.

### The effect of EEDK on the intracellular levels of ROS

**Figure 2:**
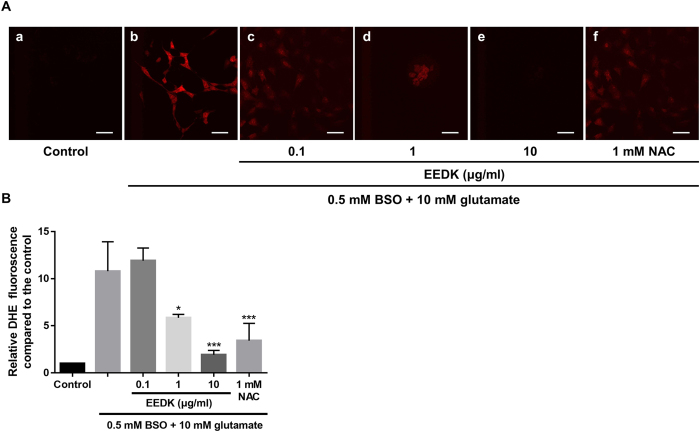
Effect of EEDK on ROS levels in response to glutamate/BSO. (**A**) Representative confocal microscopy-based fluorescence images following DHE staining (original magnification, 400×): (a) control cells (treated with 0.5% DMSO), (b) cells treated with 10 mM glutamate and 0.5 mM BSO, (c–e) cells treated with EEDK (0.1 to 10 μg/ml), glutamate, and BSO, and (f) cells treated with 1 mM NAC, with glutamate and BSO. (**B**) Quantification of DHE fluorescence was performed by analysis of 4 representative fields from each of 4 wells. Scale bar = 50 μm. The results shown are the mean values with error bars indicating the S.E.M. (*p < 0.05, ***p < 0.001). Experiments were repeated 3 times, independently.

DHE staining was performed to evaluate the efficacy of EEDK in blocking superoxide production induced by glutamate/BSO treatment. DHE has been extensively used in cell or tissue culture to assess ROS production^40^,^41^. DHE can permeate the cell membrane and react with superoxide anions, which eventually forms a red fluorescent product upon DNA intercalation. As shown in Fig. 2, when the cells were exposed to glutamate/BSO, red fluorescence increased by approximately 11-fold (relative DHE fluorescence 10.78 ± 3.1), compared with that observed in control cells. However, DHE-positive cells were significantly diminished following EEDK treatment in a concentration-dependent manner, with a 10 μg/ml EEDK pre-treatment showing the highest potency (DHE fluorescence 1.92 ± 0.5 relative to control). This inhibitory effect was even stronger than that observed following treatment with 1 mM NAC, which was used as a positive control (Fig. 2B). Thus, our data demonstrated that EEDK can strongly reduce excessive intracellular ROS production.

### Antioxidant activities of EEDK

To verify that the protective properties of EEDK against glutamate/BSO-induced cell death might be due to antioxidant activities, western blot analysis was performed to assess changes occurring in antioxidant protein levels. GST has been well documented to serve antioxidant roles following cytotoxicity and stress-induced apoptosis^42^,^43^. GST is broadly expressed in retinal cells such as RGCs and retinal pigment epithelial cells, where it could become activated upon intracellular ROS accumulation to catalyse glutathione conjugation with substrates *via* the sulfhydryl group, resulting in detoxification or the prevention of oxidant-induced cellular damage^44^,^45^.

**Figure 3:**
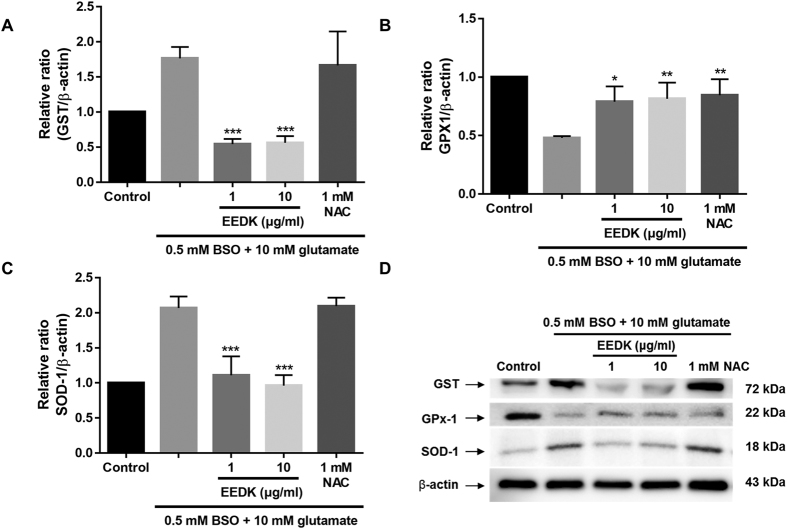
Effect of EEDK on antioxidant-associated proteins. (**A**–**C**) Bar graphs showing quantitative analysis of the corresponding proteins. (**D**) Western blot analysis of the effects of EEDK pre-treatment on expression of the antioxidant-associated proteins GST, GPx-1, and SOD-1. Protein levels are expressed as the mean ± S.E.M. from 3 independent experiments (*p < 0.05, **p < 0.01, and ***p < 0.001).

In fact, glutamate/BSO treatment elevated GST expression in RGC-5 cells, while EEDK pre-treatment blocked GST induction (Fig. 3A). A different type of antioxidant enzyme (SOD-1) was also tested. This enzyme appears to be capable of scavenging free superoxide radicals in the retina^46^,^47^ and catalysing a reaction with the superoxide radical to generate ordinary oxygen (O_2_) or hydrogen peroxide (H_2_O_2_)^46^,^47^. We observed that SOD-1 exerted similar antioxidant activity to GST (Fig. 3B). Conversely, inactivation of glutathione peroxidase-1 (GPx-1) was reported in response to various sources of oxidative stress, which promote the accumulation of peroxides^48^. As expected, glutamate/BSO treatment lowered the protein level of GPx-1, whereas cells treated with glutamate/BSO, and EEDK showed significantly increased GPx-1 protein levels (Fig. 3C,D, and Supplementary Fig. 2). These results indicated that EEDK could protect RGC-5 cells from oxidative stress-induced retinal damage by modulating antioxidant enzyme activities.

### EEDK mediates expression of the apoptotic proteins PARP, p53, and caspase-3

Because the activities of antioxidant enzymes closely correlate with the expression of apoptotic proteins and signalling through such pathways^49^,^50^, we examined whether EEDK could mediate apoptotic signals at the protein level.

**Figure 4:**
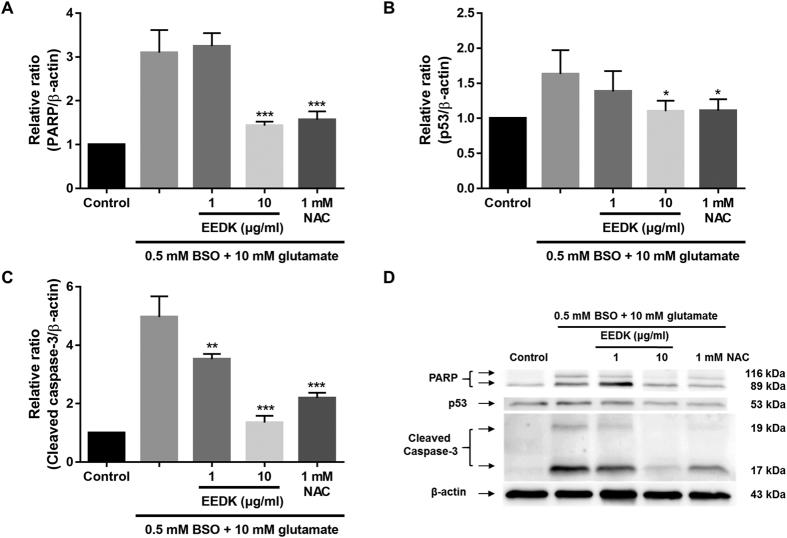
Effect of EEDK on the levels of the apoptotic proteins PARP, p53, and cleaved caspase-3. (**A**–**C**) Bar graphs showing quantitative analysis of the corresponding proteins. (**D**) Western blot analysis of the effects of EEDK pre-treatment on expression of the antioxidant-associated proteins PARP, p53, and cleaved caspase-3. Protein levels are expressed as the mean ± S.E.M. from 3 independent experiments (*p < 0.05, **p < 0.01, and ***p < 0.001).

PARP is a pivotal protein associated with DNA repair and apoptosis^51^,^52^. It has been shown that this protein can be activated in cells in response to oxidative stress and DNA damage^53^,^54^,^55^. In fact, we observed that glutamate/BSO treatment increased PARP protein expression by 3-fold compared to that observed in control cells, while EEDK treatment (10 μg/ml) significantly reduced PARP protein expression (Fig. 4A,D). Moreover, oxidative stress is likely to trigger expression of the p53 tumour suppressor protein and caspase-3, both of which are highly engaged in regulating different forms of stress responses and associated molecular networks by inducing apoptosis^56^,^57^. Similar to previous findings, under the stress condition with glutamate/BSO treatment, we found that p53, and caspase-3 protein expression were noticeably up-regulated in RGC-5 cells. However, pre-treatment with EEDK suppressed the up-regulation of these proteins in a dose-dependent manner (Fig. 4 and Supplementary Fig. 2), suggesting that EEDK may protect RGC-5 cells *via* down-regulating apoptotic proteins.

### EEDK protects the retinal layer and RGCs *in vivo*

Experimental animal models with optic nerve crush (ONC) have been well-established in a wide range of studies, in particular for glaucoma^58^. In our experiments, surgically exposed optic nerves were partially clamped short-term, which initiated RGC death, further promoting the death of uninjured, surrounding RGCs due to optic nerve injury. An experimental animal model of optic nerve crush has been suggested to serve as a model of mechanical axon injury^59^. In particular, a PONC model, an experimental procedure of a standardized and reproducible incomplete axotomy of the RGCs, mimics the key pathological features related to RGC apoptosis^60^. In our study, the exposed optic nerve was partially crushed for 7 seconds, and the survival rate of RGCs was reduced by approximately 32.3% compared with control mice, as measured by Fluoro-Gold staining.

**Figure 5:**
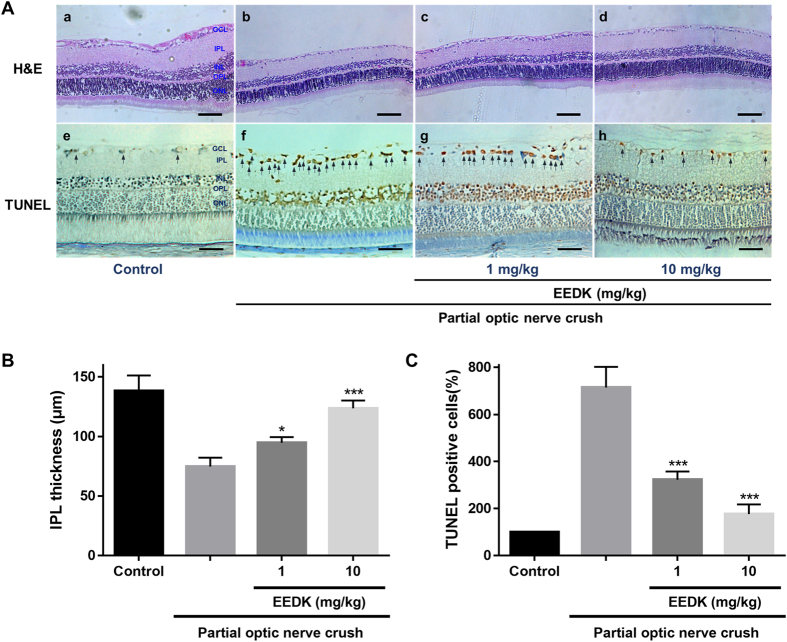
Histological analysis of retinas induced by PONC in mice. Forty mice (6-week-old males) were randomly divided into 4 groups (n = 8 mice per group), including the non-treated group (control), vehicle group (PONC only), 1 mg/kg EEDK-treated group (with PONC), and 10 mg/kg EEDK-treated group (with PONC). (**A**) Representative photomicrographs showing the histological appearance of retinal cross-sections following H&E staining and TUNEL assays. (**B**,**C**) Comparison of IPL thicknesses and TUNEL-positive cells between the EEDK-treated group and the vehicle-control group. Values are expressed as the mean ± S.E.M from 3 independent experiments (*p < 0.05 and ***p < 0.001).

Utilizing this approach combined with haematoxylin and eosin (H&E) staining and terminal deoxynucleotidyl transferase-mediated dUTP-biotin nick end-labelling (TUNEL) assays, we investigated whether EEDK could protect the retinal layer, particularly the inner plexiform layer (IPL), in a PONC-induced retinal model. This approach was taken because RGC death in the ganglion cell layer (GCL) and deterioration of its relevant layer (the IPL) are the primary by-products that result from optic nerve injury in the retina. H&E staining has long been used for morphological evaluation and to identify various tissue types^61^. In addition, TUNEL assays have been used to detect apoptotic cells in tissues^62^,^63^. We observed that the thickness of the IPL in the retina was significantly preserved after PONC in the mouse group treated with EEDK, in a dose-dependent manner, compared to the vehicle-control group (Fig. 5B). Quantification of apoptotic cells in the retinal GCL by TUNEL assays was performed in both the normal and PONC-induced groups. Control tissues exhibited relatively fewer TUNEL-positive cells, but a large number of TUNEL-positive (i.e., apoptotic) cells were counted in the GCLs of vehicle control-treated tissues. Importantly, the EEDK-administration group showed a significant decrease in the percentage of TUNEL-positive cells in the GCL (Fig. 5C). These results suggested that EEDK plays a protective role for the GCL and IPL against optic nerve injury-induced retinal degeneration *in vivo*.

### EEDK protects RGCs from PONC-induced retinal damage *in vivo* by modulating the expression of apoptotic proteins PARP, p53, and caspase-3

**Figure 6:**
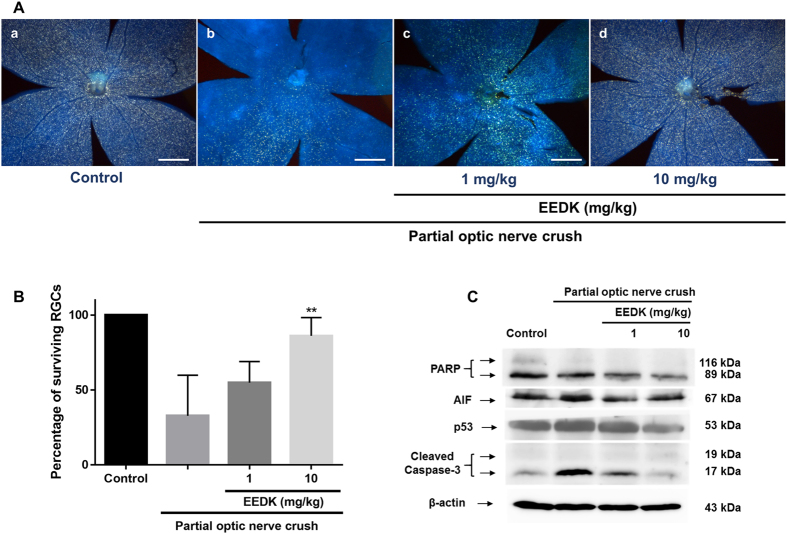
Effect of EEDK on RGC survival and expression of apoptosis-associated proteins in PONC-induced mice. Forty mice (6-week-old males) were randomly divided into 4 groups (n = 8 mice per group). (**A**) Representative fluorescence images of retrograde-labelled RGCs in PONC-induced mice: (a) control mice, (b) vehicle (PONC only), (c) 1 mg/kg EEDK-treated group (with PONC), and (d) 10 mg/kg EEDK-treated group (with PONC). Scale bar = 500 μm. (**B**) The bar graph shows quantitative analysis of the RGC survival rate (%) (**p < 0.01, mean ± S.E.M.) (**C**) Expression of apoptotic protein levels (PARP, AIF, p53, and cleaved caspase-3) in mouse retinas after damage, with or without PONC.

Neuroanatomical tracers allowing retrograde labelling of RGCs have been widely used to study RGC survival in a range of applications^64^. This approach could exclude the population of displaced amacrine cells located in the RGC layer that may impede accurate quantification in RGC survival assays. Accordingly, retrograde-labelled RGCs in flat-mount mouse retinal preparations can be evaluated through fluorescent microscopic images (Fig. 6). Retinal cell preparation was performed by injecting Fluoro-Gold dye into the superior colliculus of the brain and then assessing the cells 1 and 4 weeks after PONC. Through this assay, we observed that EEDK-administrated mice (10 mg/ml) had a higher percentage of RGC survival in comparison with that in the vehicle-control group, while in the absence of EEDK administration, the survival rate of RGCs significantly decreased (Fig. 6A,B, and Supplementary Figs 4 and 5).

To elucidate the underlying mechanisms supporting RGC survival, we performed western blot analysis against apoptotic proteins, such as PARP, AIF, p53, and cleaved caspase-3 in mouse retinal cells. Similar to our *in vitro* data, PARP protein expression decreased in EEDK-administrated retinas after PONC (Fig. 6C, and Supplementary Fig. 3).

Consistently, the expression levels of cleaved caspase-3 and p53 were suppressed by EEDK administration in PONC-induced mouse retinas (Fig. 6C). Interestingly, we observed that expression of the AIF protein, which is known as a caspase-independent death effector, was also inhibited by EEDK administration, implying the putative involvement of a caspase-independent apoptotic pathway. Taken together, our results suggested that EEDK could protect RGCs from PONC-induced retinal degeneration by regulating anti-apoptotic mechanisms and antioxidant activities.

In summary, the data generated in this study demonstrated that EEDK can exert potent protective effects on glutamate/BSO-induced RGC-5 cell death *in vitro.* Administration of EEDK can also protect RGCs from PONC-induced retinal degeneration *in vivo*. In particular, we found that this protective effect might be due to anti-apoptotic potencies in conjunction with antioxidant activities. Collectively, our findings suggest the novel possibility that EEDK is potentially an effective agent for preventing and treating retinal-degeneration diseases, such as glaucoma.

## Additional Information

**How to cite this article**: Ryul Ahn, H. *et al*. Persimmon Leaves (*Diospyros kaki*) Extract Protects Optic Nerve Crush-Induced Retinal Degeneration. *Sci. Rep.* **7**, 46449; doi: 10.1038/srep46449 (2017).

**Publisher's note:** Springer Nature remains neutral with regard to jurisdictional claims in published maps and institutional affiliations.

## Supplementary Material

Supplementary Information
