# Supplementary material for: Persimmon Leaves (Diospyros kaki) Extract Protects Optic Nerve Crush-Induced Retinal Degeneration
Source: Sci Rep. 2017 Apr 20;7:46449. doi: 10.1038/srep46449 (PMC5397840; doi:10.1038/srep46449)
Supplement: Supplementary Information [file srep46449-s1.pdf]

Supplementary Information

Persimmon Leaves (*Diospyros kaki*) Extract Protects Optic Nerve Crush-Induced Retinal Degeneration

Hong Ryul Ahn[a], Kyung-A Kim[a,b], Suk Woo Kang[a], Joo Young Lee[a], Tae-Jin Kim[a,b,c,*] and Sang Hoon Jung[a,b,*]

[a]Natural Products Research Center, Korea Institute of Science and Technology (KIST), Gangneung 25451, [b]Department of Biological Chemistry, Korea University of Science and Technology (UST), Daejeon 34113, [c]Department of Biological Sciences, Pusan National University, Busan 46241, Republic of Korea

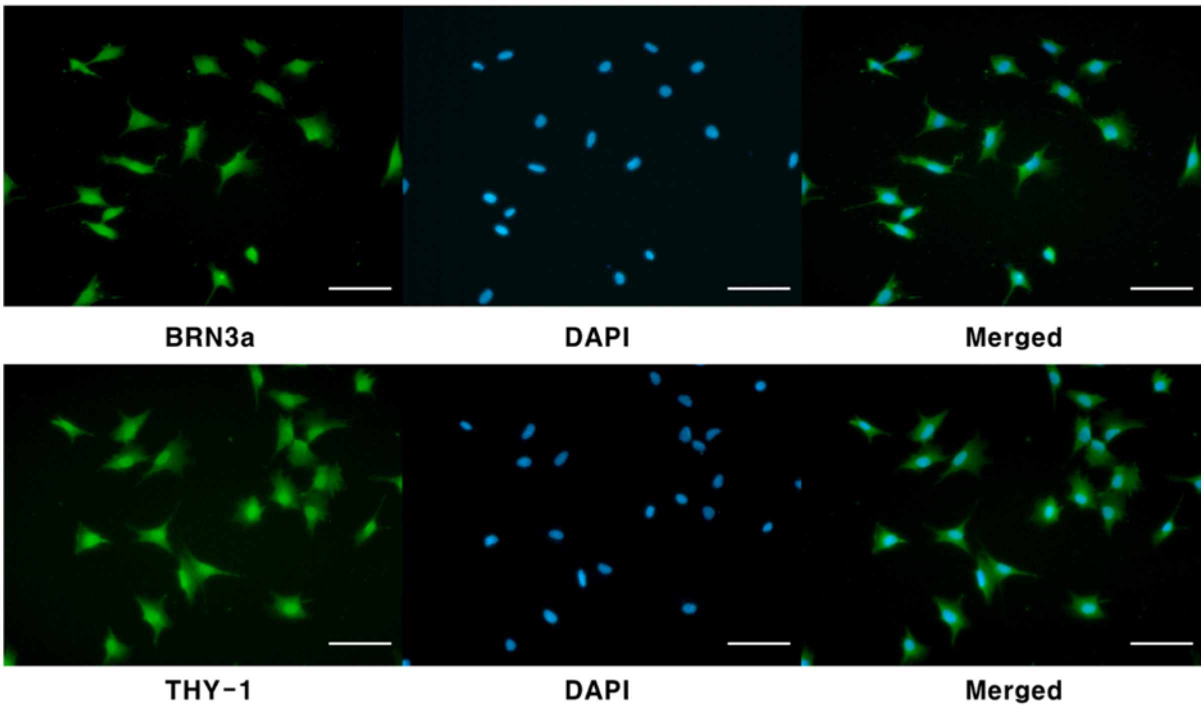

Supplementary Figure 1. Immunofluorescence of THY-1 and BRN3a proteins in RGC-5 cells. DAPI was used to visualize nuclei. Scale bar = 50 μm.

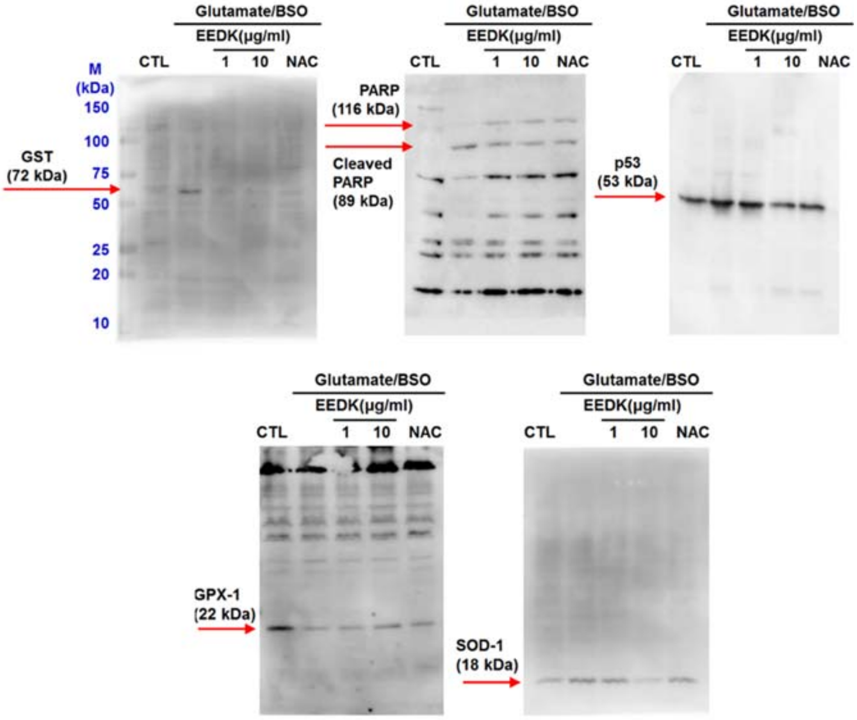

Supplementary Figure 2. Entire lanes from western blots showing the effect of EEDK on protein-expression levels in response to glutamate/BSO.

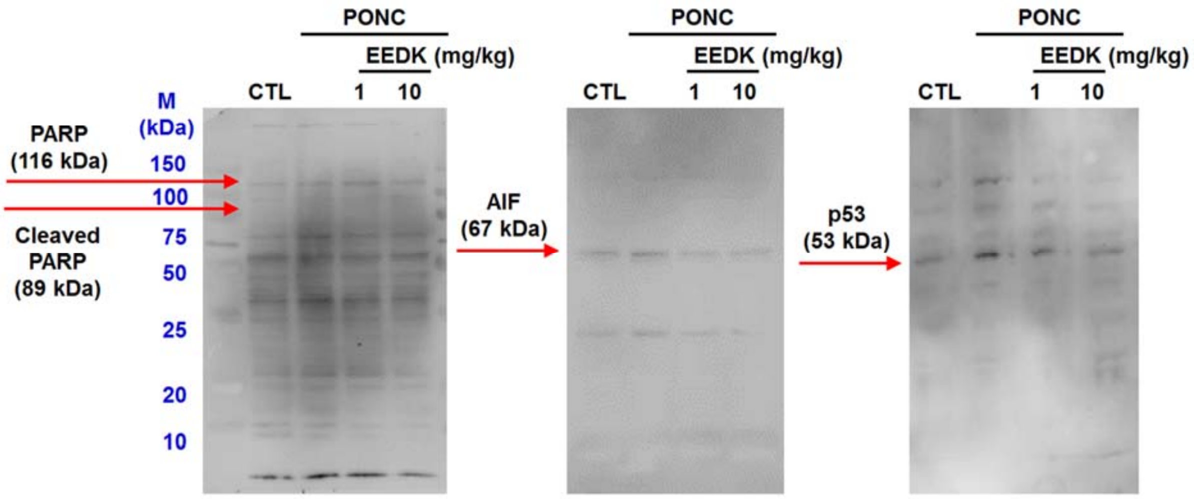

Supplementary Figure 3. Entire lanes from western blots showing the effect of EEDK on protein-expression levels in PONC-induced mice retinas.

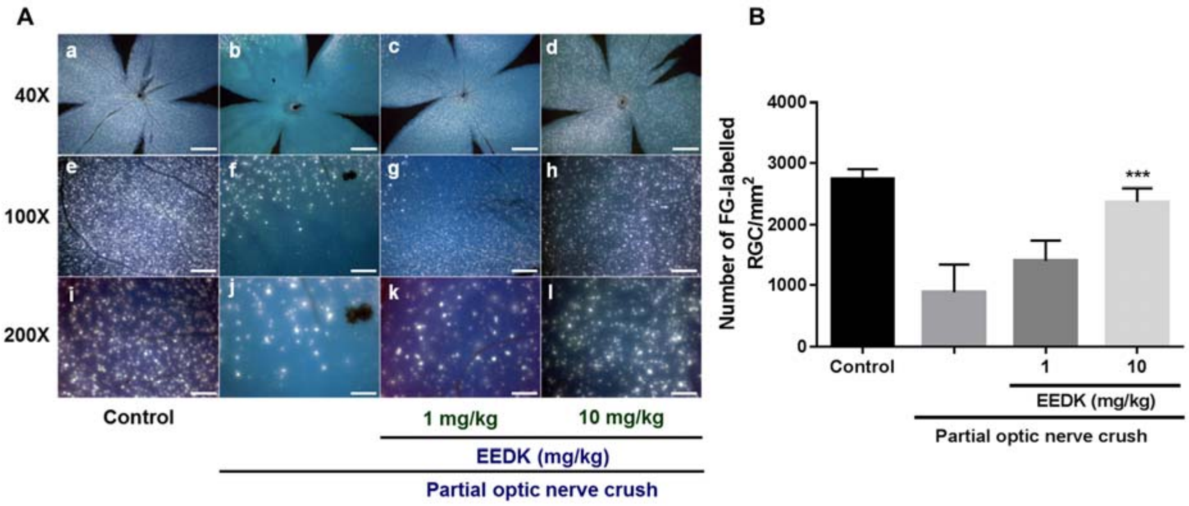

Supplementary Figure 4. Effect of EEDK on RGC survival in retinas at 1 week after PONC. (A) Representative fluorescence images of retrograde-labelled RGCs in PONC-induced mice: (a) control, (b) vehicle, (c) 1 mg/kg EEDK-treated, and (d) 10 mg/kg EEDK-treated groups. Scale bar = 500 μm. Low-magnification (×100) images of labelled RGCs in a−d are shown in e−h. Scale bars in d−f represent 200 μm. High magnification (×200) images of a−d are shown in i−l. Scale bars in i−l represent 100 μm. (B) The bar graph shows quantitative analysis of the RGC density (cells/mm^2) (***$p < 0.001$, mean ± S.E.M. n = 8 from each group).

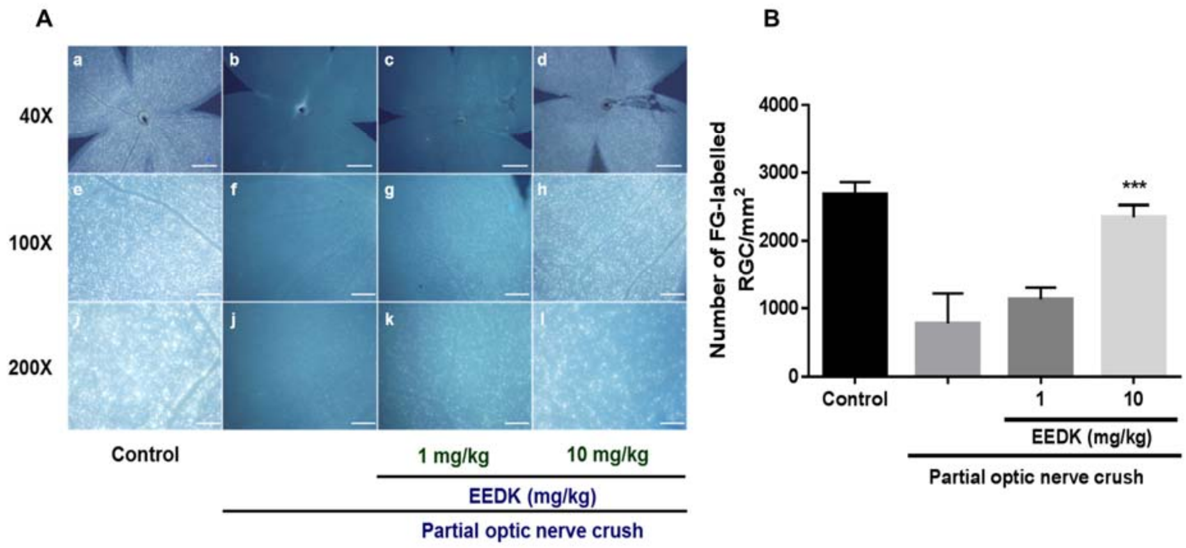

Supplementary Figure 5. Effect of EEDK on RGC survival in damaged retinas at 4 weeks after PONC. (A) Representative fluorescence images of retrograde-labelled RGCs in PONC-induced mice: (a) control, (b) vehicle, (c) 1 mg/kg EEDK-treated, and (d) 10 mg/kg EEDK-treated groups. Scale bar = 500 μm. Low-magnification (×100) images of labelled RGCs in a−d are shown in e−h. Scale bars in d−f represent 200 μm. High magnification (×200) images of a−d are shown in i−l. Scale bars in i−l represent 100 μm. (B) The bar graph shows quantitative analysis of the RGC density (cells/mm^2) (***p < 0.001, mean ± S.E.M. n = 8 from each group).
